# Ocular complications following intravitreal bevacizumab injection for retinopathy of prematurity and assessment of risk factors

**DOI:** 10.1186/s40942-020-00276-3

**Published:** 2021-01-11

**Authors:** Fatemeh Bazvand, Hamid Riazi-Esfahani, Ahmad Mirshahi, Alireza Khodabande, Hasan Khojastheh, Afsar Dastjani Farahani, Ramak Roohipourmoallai, Marjan Imani, Hooshang Faghihi, Nazanin Ebrahimi Adib, Mohammadreza Mehrabi Bahar

**Affiliations:** 1 Eye Research Center, Farabi Eye Hospital, Retina services, Tehran University of Medical Sciences, Qazvin square, South Kargar Street, Tehran, Iran; 2 Department of Ophthalmology, University of Florida, Gainesville, FL USA

## Abstract

**Purpose:**

Laser ablation of the avascular peripheral retina has been the standard method of ROP treatment. Intravitreal anti-VEGF is useful in the management of ROP patients, especially for aggressive posterior ROP. However, ocular and systemic complication after intravitreal bevacizumab was the main concern. This study aimed to investigate the treatment-related ocular and systemic complications of intravitreal bevacizumab (IVB) in patients with retinopathy of prematurity (ROP).

**Method:**

This retrospective study included neonates receiving intravitreal injections of bevacizumab (IVB) (0.625 mg) to treat ROP. Medical records of the patients were evaluated about the ocular complications after receiving IVB from 2012 to 2019. Treatment-related complications (vitreous hemorrhage, glaucoma, cataract, hyphema, corneal abrasion/opacity, and endophthalmitis), and disease-progression signs including retinal fold or stage 4 or 5 detachment were documented. Any reports of systemic events after injections were also recorded.

**Result:**

Mean gestational age and birth weight of 441 patients receiving IVB for type-1 ROP were 28 ± 2 (22–34 weeks) and 1121 ± 312 (550–2700 g), respectively. The median follow-up after treatment in all patients and patients with complications was 289.43 ± 257 days (5–1899 days) and 385.89 ± 311.59 (196–1192) days, respectively. Out of 865 eyes, 20 eyes (2.31%, 95% Clopper-Pearson Confidence Interval: 1.14–3.54%) have been affected by ocular complications. The rates of different complications included progression of retinopathy in 17 eyes (1.96%), cataracts in 2 eyes (0.23%), and vitreous hemorrhage in one eye (0.11%). No cases of endophthalmitis, thromboembolic events, or death occurred in this study. We evaluated the prevalence ratio (PR) on the multiple risk factors to determine the prediction of the complications. The existence of neovascularization of iris has the highest susceptibility to predict the complication (PR = 5.091, P-value 0.014) following by the presence of retinopathy in zone 1 of the infant’s retina (PR = 4.386, P-value = 0.010).

**Conclusion:**

The incidence rate of complications related to Intravitreal bevacizumab injection was low, which was compatible with previous studies. Bevacizumab injection seems well tolerated in most cases of ROP. Iris neovascularization and the presence of retinopathy in zone 1 were associated with a higher occurrence of complications than the absence of these risk factors.

## Background

Retinopathy of prematurity (ROP) is one of the major preventable causes of childhood blindness [1]. ROP is a proliferative disease of the immature retina, which causes impairment of vision and even blindness [2]. Parallel with the developments in neonatal care, and survival of more premature infants, the incidence of ROP cases has also increased [3]. Few modalities for treating the ROP complications include cryotherapy, laser therapy, vitrectomy in advanced cases, and recently the use of anti-vascular endothelial growth factor [4, 5].

Over the past decades, ablation of the avascular peripheral retina with laser photocoagulation has been the standard method of ROP treatment. It reduces the risk of complications associated with proliferative ROP, but it has disadvantages such as destroying large retina areas and inducing myopia [6–8]. With the advent of vascular endothelial growth factor (VEGF) inhibitors, new developments are emerging in ROP treatment. The Bevacizumab Eliminates the Angiogenic Threat of Retinopathy of Prematurity study (BEAT-ROP study) revealed a lower recurrence of posterior ROP treated with intravitreal injection versus laser photocoagulation. Thus, a new era began in the management of ROP patients, especially for aggressive posterior ROP. Using anti-VEGF, we can mitigate the side effects of cryotherapy and laser therapy, such as permanent visual field loss; we also had a rapid onset of action in comparison with the ablative treatments [9].

The vascular endothelial growth factor is essential for the infant's retinal development. Also, growth factors such as VEGF play an important role In normal retinal vascularization, which progresses from the optic nerve to ora serrata [1].

Ophthalmologists, as well as neonatologists, have expressed concerns over the potential ocular and systemic sided effects of anti-VEGF injections in ROP patients, which have been demonstrated to be detectable in serum samples after an intravitreal injection [10–13].

This study aimed to assess the rate of ocular and systemic complications following IVB injection for type-1 ROP patients and evaluate the risk factors associated with occurrence complications.

## Materials and methods

This retrospective case series was performed in Farabi eye hospital, a tertiary referral center. Medical records for patients with type 1 ROP treated with intravitreal anti-VEGF (Avastin, Genentech Inc., South San Francisco, CA, USA) between 2012 and 2019 were collected from the ROP center and pooled together for data analysis. This study was approved by the Hospital Review Committee and followed the principles of the Declaration of Helsinki. Standard laser treatment, as well as anti-VEGF benefits and complications, were fully explained to parents, and written informed consent was obtained from them as the routine protocol of ROP services of the hospital. Retinal specialists did all the examinations with ROP expertise or by fellowship-trained pediatric ophthalmologists.

Subjects were infants born between January 2012 and August 2019, with known gestational age, birth weight, and ROP examination status who had undergone IVB injection. Which included the presence in either eye of Early Treatment of Retinopathy (ETROP) Study type 1 (zone I at any stage with plus disease, zone I stage 3 without plus disease, or zone II stage 2 or 3 with plus disease) [9].

Infant demographic, medical, and funduscopic data were extracted retrospectively from the patient medical records by a certified data collector (F.B and H.RE) for the infants who had received IVB. Any related ocular complications, including corneal abrasion/ulcer, crystalline lens opacity or cataract, hyphema glaucoma, vitreous hemorrhage, and post-injection endophthalmitis or uveitis, were collected. Further, disease progression was defined as a retinal fold, worsening of the present disease, or stage 4/5 ROP. Any reports of systemic events after injections were also documented.

Exclusion criteria were refusal of a parent or guardian, any history of laser therapy, cryotherapy, or surgical procedure before IVB injection, any associated primary ocular or systemic disorders that could confound the interpretation of study results, as well as incomplete medical records.

The injection pro0cedure was done in the operation room under topical anesthesia, while the vital signs were monitored throughout the entire procedure. The eyes were prepared in a standard fashion using 5% povidone/iodine. After 5 min, a single intravitreal injection of 0.625 mg (0.025 ml) bevacizumab (Avastin, Genentech Inc., South San Francisco, CA, USA) was done through pars plicata, 1–1.5 mm posterior to the limbus in all patients. A nurse helped hold the infant during the injection. After the injection, the patients received topical Antibiotics (gentamycin or sulfacetamide 10%) for 3 days. The infants were re-evaluated after 1 day and 7 days, and then every week or biweekly based on the eye's situation of patients to document the progression of the disease and any related complications. Afterward, the examinations continued until complete vascularization of the retina was observed.

In the case of inappropriate response (lack of improvement in plus and neovascularization or progression of presence disease) to anti-VEGF treatment, including sustained vascular changes, conventional confluent laser photocoagulation of ROP was performed.

Response to IVB was defined as the disappearance or decline in retinal vessel tortuosity and dilation as well as fading of retinal neovascularization, along with the development of vascularization toward the peripheral retina.

The primary outcomes of the study were to report the rate of ocular and systemic complications such as corneal opacity, cataract or lens opacity, hyphema, glaucoma, vitreous hemorrhage, endophthalmitis, death, stroke, and cerebrovascular accident. The secondary outcome was to evaluate the rate of disease progression following IVB treatment for ROP.

### Statistical analysis

We report the data as Mean ± SD. The rate of complications was reported in prevalence. We analyze the prevalence ratio (prevalence of risk factors in complicated eyes/prevalence of risk factors in uncomplicated eyes) to determine ocular and systemic risk factors, to determine the factors to anticipate anti-VEGF associated complications. In this series, we evaluated the gender of patients, the zone of retinal retinopathy involvement in infants, presence of neovascularization in iris prior to treatment, history of intubation in hospitalization period in a neonatal intensive care unit (NICU), case of twin delivery, any history of blood transfusion at the time of hospitalization, history of intraventricular hemorrhage, history of sepsis in the hospitalization period, presence of anemia in lab tests, and history of phototherapy due to neonatal jaundice from the medical records of patients. A *P* value less than 0.05 was considered statistically significant. Statistical analysis was performed using SPSS software version 23 (SPSS, Inc., Chicago, IL).

## Results

This series included 865 eyes from 441 patients who underwent intravitreal bevacizumab injection for type 1 ROP from 2012 to 2019.

The mean gestational age of infants was 28 ± 2 weeks (22–34), and the mean birth weight was 1121 ± 312 g (550–2700). The male to female ratio was 252/189 (57.1%/42.9%).

**Fig. 1:**
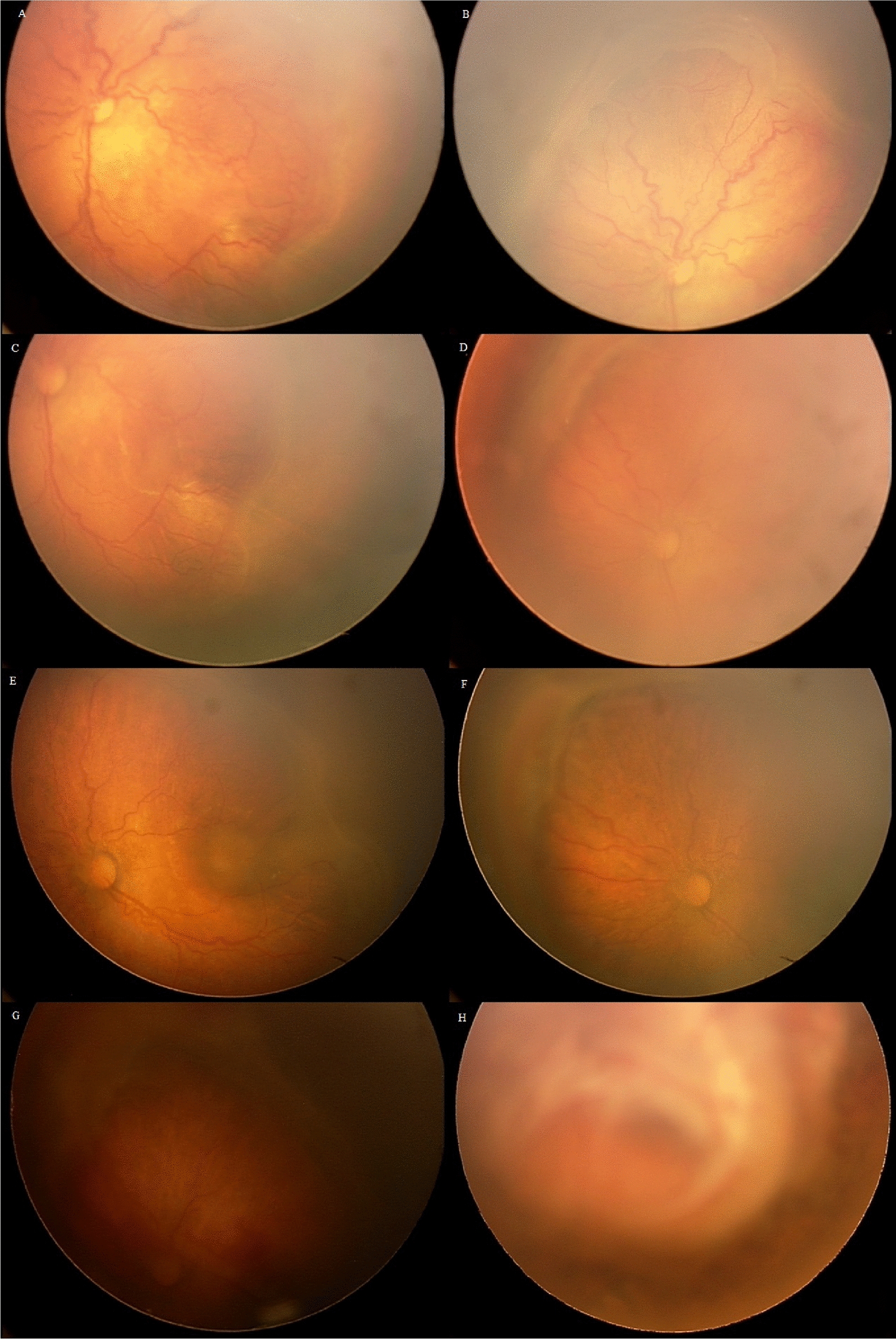
Left eye of a patient (gestational age: 28 weeks, birth weight: 1000 g) with retinopathy of prematurity (**a** and **b**: zone 1, stage3 confluent intravitreal neovascularization and plus disease). Intravitreal Bevacizumab was injected in this eye. The plus disease was improved significantly (**c**), and neovascularization also reduced. The traction increased in the supranasal part of the retina (**d**). Laser photocoagulation of the avascular area was done (**e** and **f**), and the traction increased 7 weeks after laser (**g**). Re-injection of intravitreal Bevacizumab was performed. Parents of the patient have not accepted vitrectomy. Finally, the crunch phenomenon occurred (**h**)

We observed 20 complicated eyes from 12 patients (male/female: 4/8) out of 865 eyes (2.31%, 95% Clopper-Pearson Confidence Interval: 1.14–3.54%) with IVB injection. The complications included a progression of retinopathy in 17 eyes (1.96%), including a case with crunch phenomena (Fig. 1), cataract in 2 eyes (0.23%), and vitreous hemorrhage in one eye (0.11%). From 2 patients with localized cataract, lensectomy was required in 80 and 107 days after the detection, respectively, due to progression to complete mature cataract. Vitreous hemorrhage occurred in 1 eye, but the hemorrhage spontaneously resolved without any intervention. No post-injection endophthalmitis was reported among these cases. Detailed patient complications are described in Table 1. We observed no systemic complications in our series.

**Table 1:** The characteristics and type of complications in complicated eyes treated for retinopathy of prematurity by intravitreal Bevacizumab

Eye number	GA (weeks)	BW (grams)	Gender	Time of first treatment (days after birth)	Zone of ROP	Stageof ROP	Presence of NVI	Complications
1	27	850	M	73	1	3	No	Visually lost eye
2	27	850	M	73	1	3	No	Visually lost eye
3	26	800	F	95	2	3	No	Visually lost eye
4	26	800	F	95	2	3	No	Visually lost eye
5	29	1600	M	66	1	3	Yes	Progression to stage 4A
6	29	1600	M	66	1	3	Yes	Progression to stage 4A
7	28	1000	F	59	2	3	No	Progression to stage 4A and 5
8	28	1360	M	38	2	3	No	Cataract
9	30	1550	F	77	2	3	No	Progression to stage 4A
10	30	1550	F	77	2	3	No	Progression to stage 4A
11	26	930	F	61	2	3	No	Cataract
12	29	1200	F	42	1	3	No	Progression to stage 4A and 5
13	29	1200	F	42	1	3	No	Progression to stage 4A and 5
14	27	1000	F	57	1	3	No	Progression to stage 4A
15	27	1000	F	57	1	3	No	Progression to stage 4A
16	28	1300	F	45	1	3	Yes	Progression to stage 4A and 5
17	28	1300	F	45	1	3	Yes	Progression to stage 4A
18	25	895	M	70	1	3	Yes	Vitreous hemorraghe
19	28	1300	F	74	2	3	No	Progression to stage 4A
20	28	1300	F	74	2	3	No	Progression to stage 4A

*GA* gestational age, *BW* birth weight, *ROP* retinopathy of prematurity, *NVI* iris neovascularization, *M* male, *F* female

We evaluated the prevalence ratio (PR) on the gender of patients, the zone of the ROP in infants, presence of neovascularization in iris prior to the treatment, history of intubation in hospitalization period in NICU, case of twin delivery, any history of blood transfusion at the time of hospitalization, history of intraventricular hemorrhage, history of sepsis in the hospitalization period, presence of anemia in lab tests, and history of phototherapy due to neonatal jaundice (Table 2).

**Table 2:** The prevalence ratio (PR) of risk factors for complications

Risk factor	PR	P value
Laterality (OD/OS)	1.001	0.995
Gender (male/female)	0.321	0.073
Zone (1/2)	4.386	0.010
NVI	5.091	0.014
Intubation	1.224	0.732
Twin	1.040	0.954
Transfusion	0.950	0.931
IVH	1.460	0.714
Sepsis	1.542	0.464
Phototherapy	2.013	0.313
Anemia	0.542	0.553
ARDS	1.362	0.607

*NVI* iris neovascularization, *IVH* intraventricular hemorrhage, *ARDS* acute respiratory distress syndrome

In our study, 54 eyes (6.2%) had neovascularization of iris before the administration of anti-VEGF, and among them, five eyes (9.3%) had at least one complication from the aforementioned complications. On the other hand, out of 811 eyes (93.8%) without iris neovascularization, 15 eyes (1.8%) showed complications related to anti-VEGF administration. The presence of neovascularization of iris has the highest susceptibility to predict the complication (PR = 5.091, P-value 0.014).

Retinopathy of prematurity occurred in zone 1 in 187 patients (21.6%) and zone 2 in 678 patients (78.4%). The complication rate in zone 1 ROP and zone 2 ROP following the treatment was 11 eyes (5.9%) and 9 eyes (1.3%), respectively. The presence of retinopathy in zone 1 of the infant's retina had a high chance of causing complications (PR = 4.386, P-value = 0.010). On the other hand, iris neovascularization and disease in zone 1 were associated with 5 and fourfold greater occurrence of complications as compared with the absence of these risk factors.

## Discussion

The current study revealed that among 865 eyes of 441 immature patients treated with intravitreal bevacizumab, the rate of all complications directly attributable to IVB injection for ROP was low (< 2.5%). From different documented complications, the progression of retinopathy was the main complication (2%) observed in our study. We also investigated the risk factors of complications related to IVB injections in ROP patients.

As found in this study, the risk of IVB related ocular complications was low. Based on a secondary analysis on the Postnatal Growth and Retinopathy of Prematurity study data (G-ROP), the rate of ocular complications following IVB injection was very low (1 case of vitreous hemorrhage and no case of ROP progression) [14]. Although the G-ROP study was a large multicenter retrospective study to compare the effects of IVB and laser photocoagulation in the treatment of ROP patients, during the period between 2006 and 2012 [15], the study was not powerful enough to detect the rate of the ocular complications or disease progression in eyes that had been treated with IVB. The use of IVB for ROP was not widespread during that period, as demonstrated by the small number of eyes treated with IVB (41 eyes) [15] . In this study, we had far more enrolled eyes; therefore, the rate of ocular complications following IVB could be estimated more precisely.

In the BEAT-ROP study, the retreatment rate due to ROP progression was 4% after IVB injection in infants with zone I or posterior zone II disease. They did not report any cataract, vitreous hemorrhage, or endophthalmitis in their cases [5].

A systematic and meta-analysis was done on the complications of anti-VEGF administration in ROP patients by Pertl et al. [16]. They included 24 studies with 1457 eyes. The main anti-VEGF agents used in the enrolled studies were bevacizumab, followed by ranibizumab in two and aflibercept in one trial. There were different doses of bevacizumab in the trials, from 0.25 to 1.25 mg. From 882 eyes, 55 eyes (6.2%) developed a treatment-required ocular complication, which was compatible with ROP progression in all of them, including recurrent retinal neovascularization, retinal detachment (progression to stage 4 or 5), macular fold or dragging, and persistent plus disease. Ocular complications without need for retreatment occurred in 11 cases (1.2%), including spontaneous resolved retinal hemorrhage, cataract, mild macular traction, and exotropia [16].

We also revealed the ROP progression in near 2% of our cases. Persistent areas of retinal avascularity are a concern in patients treated with IVB, which may lead to VEGF rise and the ROP progression. We observed that patients with zone 1 of ROP had a higher chance of developing ocular complications, including the ROP progression (prevalence ratio for zone 1 ROP = 4.386, P-value = 0.010). Further, we also showed that patients with Iris neovascularization, as an indicator of the severity of ischemia, had a higher chance of developing ROP progression and complications (prevalence ratio for NVI = 5.091, P-value 0.014). Thus, ROP progression may be related to baseline ROP severity. An improper patient selection for receiving IVB might be regarded as another reason. We found a crunch phenomenon in one patient. Eyes with any amount of tractions might not be suitable for anti-VEGF therapy.

We observed 2 cases with cataract and 1 case with vitreous hemorrhage among 865 the eyes treated with bevacizumab. There was no case with post-IVB corneal opacity, glaucoma, and endophthalmitis. The rate of cataract, vitreous hemorrhage and endophthalmitis was very low in our study, which was consistent with the aforementioned studies. Hwang et al. reported similar rates of vitreous hemorrhage in eyes treated with IVB [17]. The cause of post-IVB vitreous hemorrhage was unclear based upon a retrospective review of the medical records in our cases, as well as the mentioned studies.

Also, any potential benefit related to lower ocular complications must be weighed against the risk of systemic adverse effects. In an animal study of bevacizumab in rabbit eyes, the detectable level of bevacizumab in blood was shown in 8 days after administration [18]. Infants treated with IVB had systemic VEGF suppression for even seven weeks [11] after the treatment. It is speculated that systemic suppression of VEGF could hinder vascularization of developing brain, lung, kidney, or other tissues, which could lead to developmental issues associated with this treatment. [13, 19, 20].

In the BEAT-ROP study, they reported four mortalities due to lung disease in the bevacizumab group compared to one death in the laser group; although non-significant with a very small number of events, it raised concern over pulmonary maturation arrest due to VEGF blockade. [5] Another trial on 15 premature infants, eyes randomized into two groups, revealed no evidence of MRI finding changes at 1-year follow-up or systemic adverse effects attributable to bevacizumab therapy at five years of follow-up. [21] In a systematic review of studies on systemic complications after anti-VEGF for ROP, they reported 8 patients with systemic complications (1.4%) out of 585 ROP patients. All of them had received bevacizumab for treatment. None of the complications were considered to be caused by anti-VEGF agents based on the investigators' evaluations [16]. We observed no systemic complications in our series, including cerebrovascular accident, lung disease, thromboembolic findings, or death.

Nevertheless, the lack of evidence on long-term safety outcomes of anti-VEGF therapy is still a significant concern and needs further investigation. Importantly, a study that used the data from the Canadian Neonatal Network, after adjusting for main confounders such as gestation, gender, maternal education, Score for Neonatal Acute Physiology II (SNAP-II) score, bronchopulmonary dysplasia, sepsis, and severe brain injury, demonstrated 3.1 times higher odds (95% CI 1.2–8.4) of severe neurodevelopmental disabilities in preterm infants born before 29 weeks of gestation and treated with bevacizumab [22].

There are some case reports on the rare but significant complications of intravitreal anti-VEGF use in ROP patients. Lee et al. reported three patients with fibrous retinal traction band, which occurred after an intravitreal bevacizumab injection for stage 3 ROP with plus disease [23]. Chhablani et al. described a case with choroidal ischemia secondary to an intravitreal bevacizumab injection for the treatment of AP-ROP [24]. Wu et al. reported twin preterm infants who developed hypotension associated with intravitreal bevacizumab therapy for ROP [25]. Recently, Twitty G et al. reported a 25-week premature infant, treated with bevacizumab for stage 3 ROP and developed systemic hypertension after administration. Also, they observed neuroimaging changes as vasogenic edema, which improved over time [26].

Strengths of our study included the large number of IVB-treated eyes; All patients were treated at the same center, which used standardized treatment protocols. Our study had some limitations as well. First, the study had a retrospective design. Secondly, some of the infants treated with IVB primarily were subsequently treated with laser due to disease progression; therefore, some late complications of IVB injection may be precluded. Further, some delayed developmental complications of the IVB injections were not evaluated as all patients were not followed until complete vascularization was reached. Moreover, the effect of reduced doses of bevacizumab on the ocular and systemic side effects was not evaluated in this study; though many studies have investigated different doses of anti-VEGF for ROP patients, systemic absorption remains a major concern [9, 16]. Finally, an absence of disease progression alone may not be an adequate predictor of final visual function. Based on a randomized controlled trial, all eyes treated with IVB demonstrated peripheral and macular vascular abnormalities on fluorescein angiography 9 months after the treatment, while the majority of laser-treated eyes did not demonstrate these abnormalities [27]. Generally, a well-designed prospective study is necessary to more precisely describe the ocular complications and disease progression rates following IVB for ROP as well as to assess the potential systemic and developmental effects of the IVB injections in infants.

## Conclusion

the incidence rate of complications related to Intravitreal bevacizumab injection was low (2.31%), which was compatible with previous studies. Disease progression seems to be the most common concern after IVB injection in ROP patients. Infants with Zone 1 of ROP or iris neovascularization before anti-VEGF therapy tended to show a higher ocular complication rate, which can be explained with greater disease severity in these patients. We observed no systemic complications in our series.

## Data Availability

The datasets analyzed during the current study are available from the corresponding author on reasonable request.

## Abbreviations

ROP: Retinopathy of prematurity
IVB: Intravitreal bevacizumab
VEGF: Vascular endothelial growth factor
BEAT-ROP study: Bevacizumab Eliminates the Angiogenic Threat of Retinopathy of Prematurity study
ETROP study: Early Treatment of Retinopathy study
